# The Role of Serum MicroRNA-6767-5p as a Biomarker for the Diagnosis of Polycystic Ovary Syndrome

**DOI:** 10.1371/journal.pone.0163756

**Published:** 2016-09-27

**Authors:** Do Kyeong Song, Yeon-Ah Sung, Hyejin Lee

**Affiliations:** Department of Internal Medicine, Ewha Womans University School of Medicine, Seoul, Korea; Zhejiang University College of Life Sciences, CHINA

## Abstract

**Background:**

Polycystic ovary syndrome (PCOS) is a heterogeneous disorder, and the underlying molecular mechanisms are not clear. To date, few studies have been conducted on the altered expression of serum microRNAs (miRNAs) in women with PCOS. The present study was performed to examine the role of the serum miRNA as a biomarker for the diagnosis of PCOS and its relationship with metabolic and reproductive traits.

**Methods:**

A cross-sectional comparison was made in 21 women with PCOS and age- and body mass index (BMI)- matched 21 healthy women in an academic center laboratory between December 2008 and October 2010. We selected miRNAs that were more than 1.5-fold up-regulated or less than 0.67-fold down-regulated in women with PCOS compared with controls using the SurePrint G3 Human miRNA Microarray. Subsequently, we validated the relative expression of the miRNAs using TaqMan quantitative real-time polymerase chain reaction (RT-qPCR) assays.

**Results:**

Serum miRNA-4522, miRNA-324-3p, and miRNA-6767-5p were down-regulated in women with PCOS compared with controls in the microarray analysis. Among these miRNAs, serum miRNA-6767-5p was validated (fold change in women with PCOS/controls = 0.39, *P*-value<0.05) by RT-qPCR. The miRNA-6767-5p was negatively associated with fasting glucose (β = -0.370) and positively associated with the number of menses per year (β = 0.383) after adjustment for age and BMI (*P*s<0.05). Genes targeted by miRNA-6767-5p were involved in the cell cycle and the immune system.

**Conclusions:**

Serum miRNA-6767-5p may be a novel candidate as a molecular biomarker in the diagnosis of PCOS and may participate in the development of the metabolic and reproductive traits of PCOS.

## Introduction

Polycystic ovary syndrome (PCOS) is a common female endocrinopathy and affects 5–10% of reproductive-age women [1, 2]. PCOS is a heterogeneous disorder with a broad spectrum of phenotypes. It is characterized by ovulatory and menstrual dysfunction, hyperandrogenism, and polycystic ovaries. In addition, PCOS is associated with metabolic abnormalities, such as obesity, insulin resistance, and dyslipidemia [3]. Although the pathogenesis of PCOS has not been fully elucidated, abnormal folliculogenesis and gonadotropin production contributes to the development of PCOS. These abnormalities may arise from both genetic predisposition and environmental insults [4]. However, the underlying molecular mechanisms are not clear.

microRNAs (miRNAs) are small, noncoding single-stranded RNA sequences of 21–25 nucleotides in length and negatively regulate gene expression at the post-transcriptional level by influencing the evolution and stability of messenger RNAs (mRNAs) [5]. miRNAs are involved in the regulation of various developmental and physiological processes, including cell proliferation, differentiation, apoptosis, and hormone biosynthesis and release [6]. These short RNA molecules are conserved in various species from worms to mammals [7]. Recently, miRNAs have been highlighted in the field of disease biomarkers [8, 9]. Extracellular miRNAs circulate freely in the blood, and measurements of circulating miRNAs have the advantages of stability and easy detection in plasma or serum [9]. Therefore, blood miRNA may serve as a noninvasive biomarker for the diagnosis of various diseases and lead to a greater understanding of the molecular mechanisms underlying the pathogenesis of diseases. For example, miRNA expression profiling of human tumors has been associated with diagnosis, staging, progression, prognosis, and response to treatment [10].

Several studies on the altered expression of miRNAs have been reported in women with PCOS. The expression of whole blood miRNA-21, miRNA-27b, miRNA-103, and miRNA-155 was significantly increased in obese women with PCOS compared with lean women with PCOS. Serum free testosterone levels were positively associated with the expression of whole blood miRNA-21, miRNA-103, and miRNA-155 [11]. Although serum miRNA-222, miRNA-146a, and miRNA-30c were suggested as novel biomarkers for the diagnosis of PCOS [12], few studies have been conducted on the altered expression of serum miRNAs in women with PCOS [13, 14]. The aim of this study was to examine the role of serum miRNA as a biomarker in the diagnosis of PCOS and its relationship with metabolic and reproductive traits of PCOS in Korean women.

## Materials and Methods

### Subjects

We conducted a two-stage study to identify biomarkers for PCOS. The initial study population consisted of 8 PCOS cases and 9 controls. The second validation study included 21 PCOS cases and 21 age- and body mass index (BMI)- matched controls. The volunteers included women with menstrual irregularity and healthy women as control subjects who were recruited by local advertising at the Endocrinology and Gynecology Clinics of Ewha Womans University Mokdong Hospital. The diagnosis of PCOS was based on the National Institute of Health criteria as follows: (1) amenorrhea or oligomenorrhea (< 8 menstrual cycles per year) and (2) clinical or biochemical hyperandrogenism [15]. Clinical hyperandrogenism was defined as the presence of hirsutism with a modified Ferriman-Gallwey score ≥ 3 [16]. Biochemical hyperandrogenemia was defined as a total testosterone level or free testosterone level above the 95^th^ percentile (total testosterone > 67 ng/dl or free testosterone > 0.84 ng/dl) based on the testosterone levels in healthy women with regular menses as described in detail previously [17, 18]. Patients with similar clinical presentations, such as those with congenital adrenal hyperplasia, androgen-secreting tumors, and Cushing’s syndrome, were excluded from the study. Control subjects had no evidence of ovulatory dysfunction or hyperandrogenism. Subjects were excluded if they had taken medication (e.g., steroids, oral contraceptives, metformin, or thiazide diuretics) within the past 3 months.

The institutional review board of Ewha Womans University Mokdong Hospital approved this study. And written informed consent was obtained from all participants.

### Methods

The height and weight were measured in all the subjects, and BMI was calculated as weight (kg)/height (m)^2^. A venous blood sample was obtained from each subject after an overnight fast of at least 8 hours on the third day of the menstrual cycle. In women with amenorrhea, the blood samples were obtained on a random day, and we assessed the serum progesterone levels. The women with progesterone levels < 4 ng/ml were considered anovulatory. We measured total testosterone levels via the chemiluminescent immunoassay method using a commercially available kit (Siemens, New York, NY, USA), and the mean inter-assay and intra-assay coefficients of variability (CVs) were 4.4% and 6.2%, respectively. We measured sex hormone-binding globulin (SHBG) levels by an immunoradiometric assay using a commercially available kit (DPC, Los Angeles, CA, USA; mean inter- and intraassay CVs were 7.9% and 5.3%, respectively). We calculated free testosterone levels using the formula available on the International Society for Study of the Aging Male (ISSAM) website, which is based on the total testosterone, SHBG, and albumin levels in the same sample from each subject [19]. We calculated the free androgen index as testosterone (in nanomoles per liter)/SHBG (in nanomoles per liter) × 100.

We performed the standard 75-g oral glucose tolerance test (OGTT) in the morning after an overnight fast. After 30 min of supine rest, venous blood samples were drawn at baseline and 90 and 120 min after the 75-g glucose load. We measured the plasma glucose levels via the glucose oxidase method (Beckman Model Glucose Analyzer 2, CA, USA), and the insulin levels by a radioimmunoassay using a commercially available kit (BioSource, Nivelles, Belgium). Insulin sensitivity was assessed using the insulin sensitivity index (ISI)_est_, which was significantly correlated with the insulin sensitivity index from euglycemic hyperinsulinemic clamps in young Korean women with PCOS [20]. ISI_est_ was calculated using the OGTT values [ISI_est_ (μmol/kg·min)· (pmol/L)⁻¹ = 0.157–4.576 × 10⁻⁵ × I120–0.00519 × G90–0.000299 × I_0_] [21].

Ultrasound examinations were performed with a 7-MHz transvaginal (or transrectal for virgin women) transducer (Logic 400 General Electric, Milwaukee, WI, USA). Ovarian volume was calculated according to a simplified formula for an ellipsoid (0.5 x length x width x thickness) [22]. Ovarian volume was defined as the average volume of both ovaries, and the ovarian follicle number was defined as the average number of follicles in each ovary.

Total RNA was extracted from 200 μl of serum samples using the Exosomal miRNA Purification kit (Genolution, Seoul, Korea). Synthetic *C*. *elegans* miRNAs were spiked into serum and used as an endogenous control probe [9]. The concentrations and purities of RNA were estimated using a ND-1000 NanoDrop spectrophotometer (NanoDrop). The ratio of the absorbance at 260 nm to the absorbance at 280 and 230 nm was used as an indication of sample purity, and values of 1.7–2.0 were considered indicative of relatively pure RNA. The integrity of total RNA was estimated using an Agilent Technologies 2100 Bioanalyzer.

In the discovery state, by comparing the relative expression of serum miRNAs, up- or down-regulated miRNAs were selected. We identified the expression of 2,550 miRNAs using the SurePrint G3 Human miRNA Microarray, Release 21.0 (Agilent, Santa Clara, CA, USA). Total RNA (100 ng) was treated with phosphatase and incubated at 37°C for 30 minutes. Dimethyl sulfoxide was added to the dephosphorylated RNA, followed by the application of heat and ice. Then, the dephosphorylated RNA was incubated at 16°C for 2 hours for assembly of the labeling reaction. The labeled RNA was desalted using a spin column. The desalted labeled RNA was dried with a vacuum concentrator at 45°C to 55°C for approximately 2 to 3 hours, followed by hybridization to the Agilent miRNA expression microarray at 55°C for 20 hours. Arrays were scanned using the Agilent Technologies G2600D SG12494263 microarray platform.

Complementary DNA (cDNA) corresponding to miRNAs identified from the microarray analysis was produced using the Superscript^™^ II RT-PCR System (Invitrogen, Karlsruhe, Germany) according to the manufacturer’s recommendations for oligo (dT) 20 primed cDNA synthesis. The cDNA synthesis was performed on 500 ng of RNA at 42°C. Finally, cDNA, diluted 1:2, was used in the TaqMan quantitative real-time polymerase chain reaction (RT-qPCR). RT-qPCR was performed in a ABI PRISM 7900HT Sequence Detection System (Applied Biosystems, Foster City, Calif., U.S.A.) in 384-well microtiter plates using a final volume of 10 μl. Optimal reaction conditions were obtained with 5 μl of Universal Master Mix (Applied Biosystems, Foster City, Calif., U.S.A.) containing dNUTPs, MgCl2, reaction buffer, Ampli Taq Gold, 90 nM primer(s), and 250 nM fluorescence-labeled TaqMan probes. Finally, 2 μl of template cDNA was added to the reaction mixture. The primer/TaqMan probe combinations were designed according to each target sequence. Amplifications were performed starting with a 10-minute template denaturation step at 95°C, followed by 40 cycles at 95°C for 15 seconds and 60°C for 1 minute. All samples were amplified in triplicate, and data were analyzed with Sequence Detector software (Applied Biosystems).

The target genes of miRNAs were predicted using the MiRDB web site (http://mirdb.org/cgi-bin/search.cgi) and the TargetScan Human release 7.0 web site (http://www.targetscan.org/). The biological functions of the predicted targets were assessed using the Database for Annotation, Visualization and Integrated Discovery Bioinformatics Resources 6.7 (https://david.ncifcrf.gov/).

### Statistical analyses

Array data export processing and analysis were performed using Agilent Feature Extraction v11.0.1.1. The quality of miRNAs was checked and filtered by Flag. The signal intensity of miRNAs was logarithmically transformed and quantile-normalized to achieve a normal distribution. We calculated fold changes of the normalized signal of miRNAs between the women with PCOS and control subjects. For example, a fold change in the PCOS/control ratio of 2 indicates that the expression of miRNAs in women with PCOS is 2-fold up-regulated with respect to that in control subjects. We used the comparative threshold cycle (Ct) method for relative quantification of miRNAs [23]. The ΔCt value was determined by subtracting the average endogenous control Ct value from the individual Ct value of target miRNA. The ΔΔCt value was determined by subtracting the ΔCt of the control sample from the individual ΔCt of the PCOS sample. The fold change of the PCOS sample relative to the control sample was determined by 2^-ΔΔCt = (ΔCt of women with PCOS - ΔCt of control subjects)^. The statistical analyses were performed using the SPSS 22.0 software package for Windows (IBM Corporation, Chicago, IL, USA). The Kolmogorov-Smirnov test was used to analyze the continuous variables for normality. The quantitative variables are reported as the means ± standard deviations. The women with PCOS and control subjects with different clinical and biochemical characteristics were compared using Student’s unpaired t-test. Spearman’s rho correlation coefficient was applied to assess the correlation between differentially expressed miRNAs and the metabolic and reproductive traits of PCOS. Multiple linear regression analyses were performed to determine the independent association between the relative expression of miRNA-6767-5p and SHBG, fasting glucose, and the number of menses per year after controlling for age and BMI. A *P* value < 0.05 was considered significant.

## Results

The clinical and biochemical characteristics of the women with PCOS and control subjects are summarized in Table 1. The women with PCOS had higher values of total testosterone, free testosterone, free androgen index (FAI), fasting insulin, ovarian volume, and ovarian follicular number and lower values of SHBG, ISI, and number of menses per year compared with the control subjects (all *P* < 0.05).

**Table 1:** Clinical and biochemical characteristics of women with PCOS and control subjects.

	PCOS (n = 21)	Control (n = 21)	*P*-value
Age (years)	23 ± 4	24 ± 6	0.269
BMI (kg/m^2^)	21.7 ± 2.3	22.2 ± 2.7	0.529
Total testosterone (ng/dl)	94.7 ± 17.1	42.5 ± 10.8	<0.001
SHBG (nmol/l)	46.4 ± 21.1	101.5 ± 49.0	<0.001
Free testosterone (ng/dl)	1.43 ± 0.34	0.40 ± 0.19	<0.001
FAI	8.1 ± 3.0	1.9 ± 1.1	<0.001
Fasting glucose (mg/dl)	87 ± 8	84 ± 6	0.178
Post-load 2-h glucose (mg/dl)	100 ± 18	89 ± 21	0.091
Fasting insulin (mIU/l)	7.0 ± 2.8	3.1 ± 4.3	0.001
Post-load 2-h insulin (mIU/l)	43.2 ± 28.6	27.4 ± 22.1	0.051
ISI (μmol/kg․min)․(pmol/L)⁻¹	0.10 ± 0.02	0.11 ± 0.02	0.003
Ovarian volume (cm^3^)	11 ± 3	5 ± 1	<0.001
Ovarian follicular number	12 ± 3	6 ± 2	<0.001
Menses/year	5 ± 1	10 ± 0	<0.001

Values are expressed as means ± standard deviations.

PCOS, polycystic ovary syndrome; BMI, body mass index; SHBG, sex hormone-binding globulin; FAI, free androgen index; ISI, insulin sensitivity index; menses/year, the number of menses per year.

**Fig 1:**
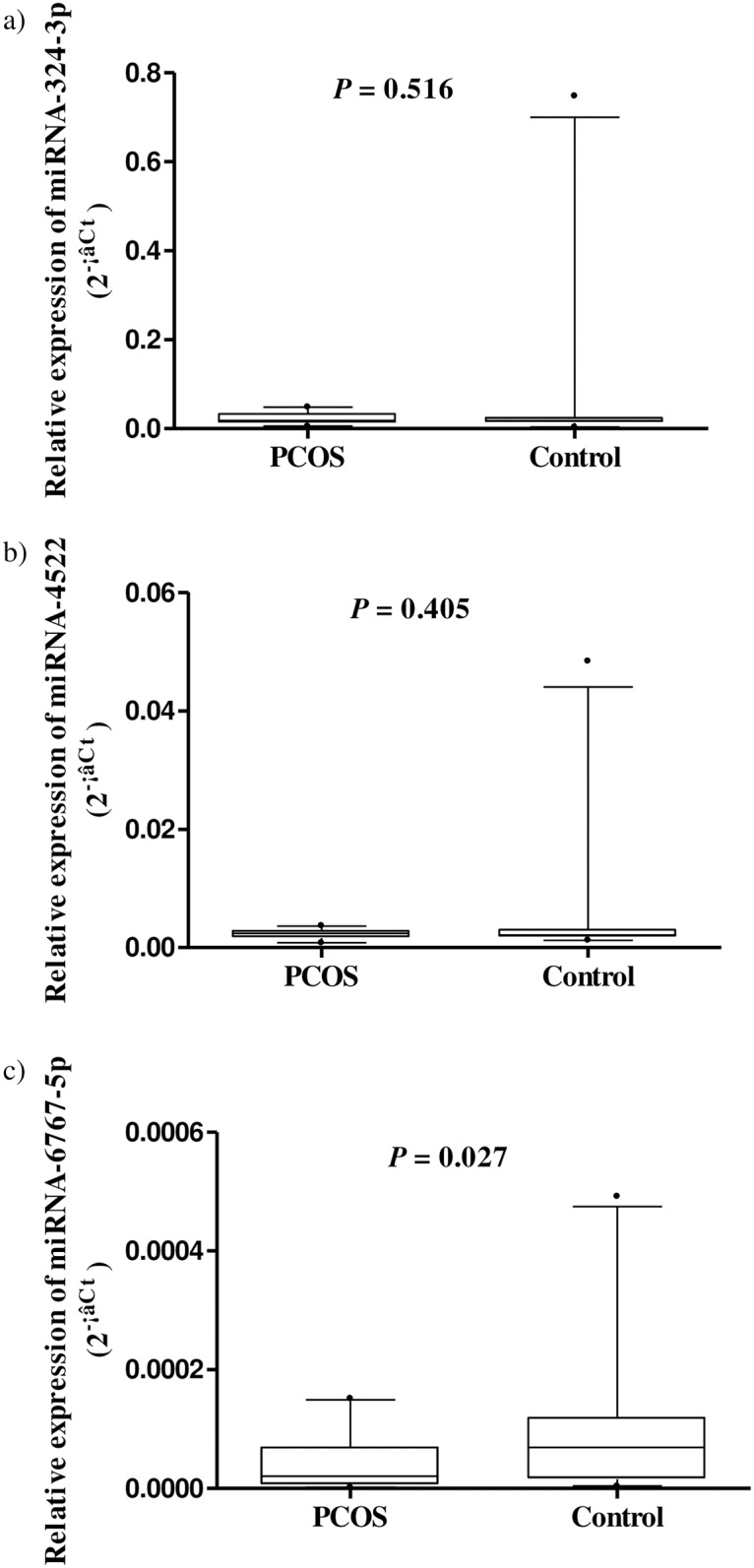
A boxplot for the relative expression of serum miRNAs in women with PCOS and control subjects from the quantitative real-time polymerase chain reaction analysis. (a-c) For each miRNA, the line inside the box is the median. The top and bottom lines of the box are the first and third quartiles, respectively. The top and bottom whiskers are the 5^th^ and 95^th^ percentiles, respectively. Any data beyond these whiskers were shown as cycles. miRNA, microRNA; PCOS, polycystic ovary syndrome; Ct, the threshold cycle.

The miRNAs that were more than 1.5-fold up-regulated or less than 0.67-fold down-regulated in the women with PCOS were identified using the microarray analysis. The expression of serum miRNA-4522, miRNA-324-3p, and miRNA-6767-5p was down-regulated in the women with PCOS compared with the control subjects (fold changes in the ratio of the expression in women with PCOS/the expression in control subjects = 0.63, 0.65, and 0.66, respectively). Among these miRNAs, miRNA-6767-5p was validated to be differentially expressed between the two groups by RT-qPCR (fold change in the ratio of the expression in women with PCOS/control subjects = 0.39, *P* < 0.05) (Table 2, Fig 1).

**Table 2:** Relative expression of serum miRNAs between women with PCOS and control subjects (2^-ΔCt^).

miRNA	PCOS (n = 21)				Control (n = 21)				PCOS/Control fold change	*P*-value
	ΔCt	Mean	SD	Median	ΔCt	Mean	SD	Median		
has-miRNA-324-3p	5.72	2.32×10^−2^	1.44×10^−2^	1.82×10^−2^	5.43	6.94×10^−2^	1.67×10^−1^	1.79×10^−2^	0.82	0.516
has-miRNA-4522	8.87	2.27×10^−3^	7.61×10^−4^	2.44×10^−3^	8.65	4.48×10^−3^	1.01×10^−2^	2.14×10^−3^	0.85	0.405
has-miRNA-6767-5p	15.63	4.19×10^−5^	4.64×10^−5^	2.08×10^−5^	14.27	9.85×10^−5^	1.21×10^−4^	6.91×10^−5^	0.39	0.027

miRNA, microRNA; PCOS, polycystic ovary syndrome; Ct, the threshold cycle; SD, standard deviation.

The relative expression of miRNA-6767-5p was positively correlated with SHBG and, the number of menses per year and negatively correlated with fasting glucose (all *P* < 0.05) (Table 3). In the multiple linear regression analysis, miRNA-6767-5p was positively associated with the number of menses per year (β = 0.383) and negatively associated with fasting glucose (β = -0.370) after adjustment for age and BMI (all *P* < 0.05) (Table 4).

**Table 3:** Correlation analysis between the relative expression of miRNA-6767-5p and reproductive/metabolic variables in total subjects.

Variables	Spearman correlation coefficients	*P*-value
Sex hormone-binding globulin	0.318	0.040
Free testosterone	-0.287	0.066
Free androgen index	-0.296	0.057
Fasting glucose	-0.357	0.020
Post-load 2-h glucose	0.143	0.367
The number of menses per year	0.365	0.017

miRNA, microRNA.

**Table 4:** Multiple linear regression analysis of relative expression of miRNA-6767-5p with reproductive and metabolic variables.

Dependent variables	R^2^	Unstandardized		Standardized	*P*-value	CI
		β	S.E.	β		
SHBG	0.211	8.417	4.869	0.252	0.092	-1.439~18.273
Fasting glucose	0.317	-1.904	0.697	-0.370	0.009	-3.314~-0.493
Menses/year	0.175	0.765	0.298	0.383	0.014	0.163~1.368

The independent variable is the logarithmically transformed 2^-ΔCt^ of miRNA-6767-5p.

Adjusted for age and body mass index.

miRNA, microRNA; SHBG, sex hormone-binding globulin; menses/year; the number of menses per year; CI, confidence interval.

The list of predicted target genes included 20 genes for miRNA-6767-5p. Genes targeted by miRNA-6767-5p participated in the cell cycle and the immune system process (Table 5).

**Table 5:** Gene ontology biological process annotations associated with target genes of differentially expressed miRNAs in women with polycystic ovary syndrome.

MiRNA	No. of target genes	Gene ontology biological processes	No. of genes	*P*-value
miRNA-6767-5p	20	regulation of cell proliferation	5	0.0053
		variable (diverse) joining recombination	2	0.0096
		somatic diversification of immune receptors via germline recombination	2	0.0228
		somatic cell DNA recombination	2	0.0228
		somatic diversification of immune receptors	2	0.0256

miRNA, microRNA.

## Discussion

In this study, serum miRNA-6767-5p was differentially expressed in the women with PCOS compared with the control subjects. The relative expression of serum miRNA-6767-5p was negatively associated with fasting glucose and positively associated with the number of menses per year. The genes targeted by miRNA-6767-5p were mainly involved in the cell cycle and immune system processes.

Since the discovery of *lin*-4 in 1993 [24], hundreds of miRNAs have been identified and the field of miRNA research has progressed dramatically. miRNAs bind to the 3’-untranslated regions of target mRNAs at multiple sites and negatively regulate target gene expression at the translational level [6]. As miRNAs can regulate several functionally related mRNAs, a single miRNA can target several genes. Moreover, a single gene may be regulated by multiple miRNAs [25]. Therefore, miRNAs play important roles in regulating developmental and physiological processes.

miRNAs can be good biomarkers of many disorders, including PCOS because the expression of miRNAs is organ-specific and related to the regulation of organ function [26]. Recently, a variety of tissue-specific expression patterns of miRNAs have been reported [6]. The Let-7 family, miRNA-21, miRNA-99a, miRNA-125b, miRNA-126, miRNA-143, miRNA-145, and miRNA-199b were the most predominant in the ovary, regardless of the species in mammals [27]. Additionally, altered miRNA expression has been reported in numerous ovarian-derived disorders, such as ovarian cancer, premature ovarian failure, and PCOS [26]. The expression of miRNA-32, miRNA-34c, miRNA-135a, miRNA-18b, and miRNA-9 was significantly increased in the follicular fluid of women with PCOS [28]. In another study, the expression of miRNA-132 and miRNA-320 was significantly lower in the follicular fluid of women with PCOS compared with control subjects [29]. The expression of miRNA-483-5p was increased in PCOS cumulus granulosa cells, and Notch3 and mitogen-activated protein kinase (MAPK)3 were demonstrated to be regulated by miRNA-483-5p [30]. Although the mechanism of the manner in which miRNAs enter the serum and whether they are biologically active are not clear; the levels of miRNAs in serum are known to be stable and can be detected directly [31]. Several studies on altered expression of miRNAs from serum or whole blood have been reported in women with PCOS. The expression of three serum miRNAs (miRNA-222, miRNA-146a, and miRNA-30c) was significantly increased in 68 Chinese women with PCOS compared with age-matched Chinese healthy control subjects [12]. In another study, 5 serum miRNAs (let-7i-3pm, miRNA-5706, miRNA-4463, miRNA-3665, and miRNA-638) were up-regulated and 4 miRNAs (miRNA-124-3p, miRNA-128, miRNA-29a-3p, and lep-7c) were down-regulated in Chinese women with PCOS compared with control subjects [32]. We measured the signals of the expression of 2,550 miRNAs, including miRNAs identified from the previous studies. The differentially expressed miRNAs in our study were different from the results of the previous studies. Except for miRNA-324-3p, the miRNAs identified in our study had not been previously detected. The expression of miRNA-324-3p was down-regulated in patients with KRAS-variant endometrial tumors [33], and miRNA-324-3p was suggested as a prognostic biomarker of endometrial cancer [34]. As obesity affects the expression of miRNAs [11], the difference in BMI could affect the different expression of miRNAs. The BMI of women with PCOS in the previous studies [12, 32] was higher (25.9–28.3 kg/m^2^) than that of women with PCOS in the present study (22.2 ± 2.7 kg/m^2^). In addition, the differences in the ethnicity of individuals with and diagnostic criteria for PCOS, the sources of miRNAs, and the manual procedures and microarrays used for identification of the expression of miRNAs could have caused the different results.

Numerous miRNAs regulate ovarian functions, such as ovarian follicular growth, atresia, and ovulation. miRNAs also regulate ovarian steroidogenesis by targeting hormone receptors and affecting hormone biogenesis and secretion [26]. Using human ovarian cells, several miRNAs were demonstrated to enhance or inhibit the release of ovarian androgen [35]. Free testosterone levels were positively associated with whole blood miRNA-21, miRNA-103, and miRNA-155, which were up-regulated in European women with PCOS [11]. In our study, the expression of miR-6767-5p was positively correlated with SHBG (*P* < 0.05), and the expression of miR-6767-5p was negatively correlated with FAI at a marginally significant level (*P* = 0.057). As hyperandrogenemia is a key characteristic of PCOS, miRNA 6767-5p may be important in the pathogenesis of PCOS. In addition, abnormal expression of miRNAs has been reported to be associated with insulin resistance and diabetes [36]. miR-93 was overexpressed in the adipose tissue of women with PCOS and women with insulin resistance [37]. The expression of miR-6767-5p was negatively associated with fasting glucose in our study. As low levels of SHBG are also a strong predictor of the risk of type 2 diabetes mellitus [38], it is possible that miRNA 6767-5p has an important role in the metabolic manifestations of PCOS.

In previous studies, the genes targeted by miRNAs differentially expressed in women with PCOS were involved in various biological processes. Genes targeted by serum miRNA-222, miRNA-146a, and miRNA-30c were involved in metastasis, the cell cycle, apoptosis, and the endocrine system [12]. The target genes of dys-regulated miRNAs in Chinese women with PCOS were involved in the immune system, adenosine triphosphate binding, MAPK signaling, apoptosis, angiogenesis, response to reactive oxygen species, and p53 signaling pathways [32]. The target genes of miRNA-32, miRNA-34c, miRNA-135a, miRNA-18b, and miRNA-9, which were up-regulated in the follicular fluid of women with PCOS, were involved in insulin regulation and inflammation [28]. The biological processes associated with miRNA-6767-5p identified in our study were not studied. The genes targeted by miRNA-6767-5p were involved in the cell cycle and immune system processes. As the proliferation and differentiation of germ cells and somatic cells are required for ovarian development [39], miRNAs associated with the cell cycle could play a fundamental role in ovarian development. The immune system is important in the pathogenesis of PCOS. Chronic low-grade inflammation was suggested to contribute to the pathogenesis of PCOS [40], and the regulation of ovarian function by macrophages, which play key roles in the immune response [41], was reported. C-reactive protein concentration, a marker of inflammation, was higher in PCOS women than the healthy control group and was correlated with BMI and decreased insulin sensitivity [42]. These findings supported the possibility that the immune system-related miRNA could involve the development of PCOS.

This is the first study to examine the aberrant expression of serum miRNAs in Korean women with PCOS. One strength of our study is that we identified a novel miRNA that had not been previously detected in women with PCOS. The other strength of our study is that we used the SurePrint G3 Human miRNA Microarray, which can identify the expression of 2,550 miRNAs, compared with only specific candidate miRNAs in the previous study, in women with PCOS [11]. The other strength of our study is the carefully selected subjects. Whereas women with PCOS were diagnosed according to the ESHRE criteria in previous studies [12, 32], a homogeneous group of classic hyperandrogenemic PCOS women according to NIH diagnostic criteria were selected in this study. It is possible that the difference in the criteria for the diagnosis of PCOS may have resulted in the identification of the novel miRNA in our study. However, the relatively small sample size of our study population could have induced selection bias. We identified that the expression of serum miRNA-6767-5p was different in Korean women with PCOS than in control subjects. miRNA-6767-5p was associated with fasting glucose and the number of menses per year. Therefore, serum miRNA-6767-5p may be a novel candidate molecular biomarker for the diagnosis of PCOS in Korean women. Further large studies are needed to validate the role of miRNA as a novel biomarker for the diagnosis of PCOS and to evaluate the functional association of miRNA with PCOS.
